# ROTEM: Multiplate monitoring in the ICU and outcome scores

**DOI:** 10.1186/cc13283

**Published:** 2014-03-17

**Authors:** U Schött, A Larsson

**Affiliations:** 1 Lund University, Lund, Sweden

## Introduction

Hemostatic disorders are common in intensive care patients and can be used as prognostic markers. The aim of this prospective clinical study was to study platelet function in intensive care patients using point-of-care viscoelastic and platelet aggregometry instruments and platelet count (PLC). Our hypothesis was that measurement of platelet function would give more information about disseminated intravascular coagulation (DIC), morbidity and mortality than PLC alone.

## Methods

Patients admitted to the ICU were monitored with ROTEM viscoelasticity and a new Multiplate platelet aggregometer; routine coagulation analyses; International Society of Thrombosis and Haemostasis and Japanese Association for Acute Medicine DIC calculated scores; Sequential Organ Failure Assessment scores, Simplified Acute Physiology Score III - Expected Mortality Rate; and real in-hospital mortality. Nonparametric tests were chosen for all statistical evaluation. *P *< 0.05 was considered significant.

## Results

A total of 128 patients with different diagnoses were studied, with 330 sampling events. Multiplate analyses correlated significantly with PLC and ROTEM. However, there were more test results below normal limits for Multiplate analyses than for ROTEM in patients with thrombocytopenia. Multiplate, ROTEM and PLC results were low in patients with high SOFA scores and in patients with overt DIC scores. These test results at admission to the ICU did not differ between survivors and nonsurvivors, and did not correlate with the length of stay in the ICU. Only EMR differed between survivors and nonsurvivors and correlated with ICU length of stay.

## Conclusion

In this study, Multiplate and ROTEM did not provide any more information than PLC about DIC, morbidity and mortality. Multiplate showed lower platelet function in more patients with low platelets than ROTEM. In patients without thrombocytopenia, all patients had normal ROTEM results, but 36% of Multiplate measurements were low. This higher sensitivity of the Multiplate to measure lowered platelet function needs to be linked to real bleeding in larger patient series to define its clinical significance.

